# Development and validation of a multi-dimensional scale to assess community health worker motivation

**DOI:** 10.7189/jogh.11.07008

**Published:** 2021-03-10

**Authors:** Ann Gottert, Tracy L McClair, Sharif Hossain, Sina Pascal Dakouo, Tim Abuya, Karen Kirk, Ben Bellows, Smisha Agarwal, Sarah Kennedy, Charlotte Warren, Pooja Sripad

**Affiliations:** 1 Population Council, Washington D.C. & New York, New York, USA; 2 Population Council, Dhaka, Bangladesh; 3 Aga Khan Foundation, Mopti, Mali; 4 Population Council, Nairobi, Kenya; 5 Johns Hopkins Bloomberg School of Public Health, Baltimore, Maryland, USA

## Abstract

**Background:**

Ensuring that Community Health Workers (CHWs) are motivated is critical to their performance, retention and well-being – and ultimately to the effectiveness of community health systems worldwide. While CHW motivation is as multi-dimensional construct, there is no multi-dimensional measure available to guide programming. In this study, we developed and validated a pragmatic, multi-dimensional measure of CHW motivation.

**Methods:**

Scale validation entailed qualitative and survey research in Mali and Bangladesh. We developed a pool of work satisfaction items as well as several items assessing the importance of hypothesized sub-dimensions of motivation, based on the literature and expert consultations. Qualitative research helped finalize scale sub-dimensions and items. We tested the scale in surveys with CHWs in Mali (n = 152, 40% female, mean age 32) and Bangladesh (n = 76 women, mean age 46). We applied a split-sample exploratory/confirmatory factor analysis (EFA/CFA) in Mali, and EFA in Bangladesh, then assessed reliability. We also gauged convergent/predictive validity, assessing associations between scale scores with conceptually related variables.

**Results:**

The final 22-item scale has four sub-dimensions: *Quality of supervision*, *Feeling valued and capacitated in your work*, *Peer respect and support*, and *Compensation and workload*. Model fit in CFAs was good, as were reliabilities for the full scale (alpha: 0.84 in Mali, 0.93 in Bangladesh) and all sub-dimensions. To construct scores for the final scale, we weighted the scores for each sub-dimension by CHW-reported importance of that sub-dimension. Final possible range was -6 to +6 (sub-dimensions), -24 to +24 (full scale). Mean (standard deviation) of full-scale scores were 5.0 (3.3) in Mali and 14.5 (5.3) in Bangladesh. In both countries, higher motivation was significantly associated with higher overall interest in their work, feeling able to improve health/well-being in their community, as well as indicators of higher performance and retention.

**Conclusions:**

We found that the Multi-dimensional Motivation (MM) scale for CHWs is a valid and reliable measure that comprehensively assesses motivation. We recommend the scale be employed in future research around CHW performance and community health systems strengthening worldwide. The scale should be further evaluated within longitudinal studies assessing CHW performance and retention outcomes over time.

Community Health Workers (CHWs) are on the front lines of improving health outcomes and health equity in communities worldwide. As critical intermediaries between the health system and the communities they serve, CHWs provide health education, deliver basic health services, conduct surveillance/reporting, and support linkages to facilities across a range of health areas including child nutrition, family planning, and antenatal and postpartum care [1,2]. Therefore, the performance and retention of CHWs is essential to the effectiveness of community health systems, which reflect a “set of local actors, relationships, and processes engaged in producing, advocating for, and supporting health in communities” as an extension of broader health systems [3].

Many CHW programs around the world have demonstrated improved health outcomes [4-7], and such programs are now roundly supported by health governing bodies. Yet challenges have emerged to achieving optimal CHW performance and retention, particularly as these programs start scaling to implementation at a national level [8]. In efforts to strengthen CHW programs, scholars have recently emphasized that “CHW programs need to move beyond an instrumentalist approach to CHWs, and take a developmental and empowerment perspective when engaging with CHWs.” [9].

Ensuring that CHWs are motivated is critical to their performance, retention, and well-being. Motivation can be defined as willingness to exert and maintain effort to achieve an organization’s goals [3,10]. Integral to CHWs’ motivation is their satisfaction with their work, that is, a pleasurable or positive emotional state resulting from the appraisal of their job or job experience [11,12]. CHWs’ motivation and satisfaction can relate to and differ by distinct dimensions of their work [9,11]. Research conducted worldwide has revealed common dimensions of CHW motivation and/or satisfaction, including perceived meaningfulness and impact of their work, having sufficient training and supplies, supportive supervision and peer relationships, feeling valued in their work, and being adequately compensated [9,13-16]. Systematically gathering evidence with this level of granularity is essential to designing and evaluating interventions to optimize CHW programs [2].

Currently there is no standardized, multi-dimensional measure of motivation among CHWs, limiting an understanding of motivation as part of countries’ community health systems strengthening efforts. For available scales, items intended to tap into motivation are often worded broadly (eg, “I feel motivated to work here”), perhaps because it is difficult to ask survey questions in ways that capture the multi-dimensional aspects of motivation. This results in general assessments of motivation with limited utility for CHW programming. In contrast, scales assessing CHW job satisfaction often do so using items related to different dimensions [11,17,18]. Yet available satisfaction scales have insufficient numbers of items per sub-dimension, suffer from poor item wording, and/or combine satisfaction-related items with other items assessing general motivation as well as organization/community commitment. Finally, scoring procedures for available satisfaction/motivation scales do not account for the fact that CHWs often value/place more importance on certain dimensions than others, which may limit these measures’ utility in predicting performance and retention [11].

In this study, we developed and evaluated a multi-dimensional scale to measure CHW motivation with CHWs in Mali and Bangladesh. The scale assesses the level of CHW satisfaction with different sub-dimensions of their work and then weights each sub-dimension by the level of importance to the CHWs to arrive at a final motivation score. This research is part of the Frontline Health Project, in which global stakeholders are developing universally-applicable measures to empirically assess the effectiveness of CHW programs [3,19-21].

## METHODS

### Study settings

In Mali, four districts in Mopti region (Mopti, Djenné, Bandiagara, Bankass) were selected for the study in collaboration with the Aga Khan Foundation which was working in that region, and in consultation with local stakeholders including the Direction National de la Santé (National Directorate of Health). CHWs in Mali provide basic community-based health services from a small building/house provided to them by the communities in which they work. They are responsible for services including behavior change communication, antenatal/postnatal home visits, management of sick children, WASH, nutrition, family planning, HIV/AIDS, tuberculosis, and malaria.

In Bangladesh, the Keraniganj upazila/sub-district of Dhaka district was selected for the study in collaboration with the Clinical Contraception Service Delivery Programme (CCSDP), Directorate General of Family Planning, Bangladesh (DGFP). This upazila/sub-district was selected due to its proximity to the capital city, Dhaka, and its categorization as a lower-performing upazila/subdistrict in terms of FP counseling and referral outcomes. In this study, we focused on two cadres of CHWs that focus on family planning: Family Welfare Assistants (FWAs), who visit households at the Union level, and Family Welfare Visitors (FWVs), who are stationed at clinics called Union Health and Family Welfare Centres (eg, facility-linked). FWAs list and mobilize pregnant women for services; counsel eligible couples on contraceptive methods and side effects; provide pills, condoms, and the second dose of injectable contraception; identify and refer couples for clinical contraception; and assist in immunizations. FWVs complete referrals they receive from FWAs; provide antenatal, postnatal, and reproductive clinical services and counseling, including IUD insertion; and immunize children.

### Scale development

We identified relevant conceptual dimensions and developed a pool of 34 items based on a review of the literature and consultation with community health experts, and in-country stakeholders. Initial dimensions identified included: *quality of supervision, feeling secure and validated in your work, growth opportunity, adaptive management and support, peer respect and support,* and *compensation*. We drafted about 4-8 items for each of these conceptual domains. Ten items were adapted from Bhatnagar’s job satisfaction scale [17]. The set of items was introduced by “How would you rate your satisfaction with the following aspects of your work?” Items were the statements like “Support your direct supervisor gives you in your work”, “Cooperation amongst CHWs” and “Amount of total financial incentives you receive.” Response options included very dissatisfied, dissatisfied, satisfied, and very satisfied.

We also created several items to assess the importance to CHWs of each dimension of their work, drawing on the content of each hypothesized sub-dimension of the work satisfaction items. The set of items was introduced by “How important to you are the following aspects of your work?” with the interviewer asked to read each statement out in full, including parentheses. An item example is “Quality of supervision (that is, your supervisor(s) actively support and value your work, and treat you respectfully and fairly)”. Response options were not very important, important and very important.

### Survey methods

We implemented the scale in surveys with 152 CHWs in Mali (January 2020) and 76 CHWs in Bangladesh (November 2019- January 2020). In both settings, we applied a census-based sampling approach in which all active and consenting CHWs in the study sites were interviewed. In Bangladesh, among these 76 CHWs, we additionally observed 1260 CHW-client interactions and conducted follow up interviews with 1384 clients about their counseling and care experience. This nested sampling in Bangladesh allowed for linking CHW surveys with observations and client surveys, which was helpful in assessing convergent validity.

Surveys were administered by trained research assistants in a private setting in the CHW’s workplace, either in the community or at a facility, and lasted around 45 minutes. In Mali, surveys were administered in French or the local language and in Bangladesh they were conducted in Bengali. Research assistants administered the survey using the Ona.io platform on ARCHOS tablets in Mali, while surveys were paper-based in Bangladesh with data entry undergoing quality checks. No compensation was provided to CHWs for participation.

### Qualitative research to assess sub-dimensions and content

We conducted eight focus group discussions (FGDs) across four sites with CHWs in Mali (with 59 total participants; January 2020), and 20 in-depth interviews (IDIs) with CHWs and supervisors in Bangladesh (October-November 2019). In Mali, all CHWs who participated in the quantitative survey were invited to participate in the FGDs. In Bangladesh, IDIs were conducted with FWAs assigned to geographic areas near 10 different UHFWCs (n = 10), FWVs working at five different UHFWCs (n = 5), and Family Planning Inspectors that supervise FWAs (n = 5). The choice of using FGDs vs IDIs, as well as means of identifying/selecting participants, was made by each country study team based on anticipated value of those methods to answering study research questions.

Study team members trained in qualitative methods facilitated the FGDs/IDIs. FGDs in Mali were conducted in a combination of local languages and French; IDIs in Bangladesh were conducted in the local language (Bengali). Interviews/discussions lasted between thirty minutes to one hour. Audio recordings were transcribed verbatim and translated into English. Data were analyzed using content analysis. Coding was performed in NVivo 12 software using a codebook based on hypothesized sub-dimensions motivation, as well as related emergent themes.

Qualitative findings generally supported the relevance of each of the sub-dimensions covered in the scale items on the surveys, and often also the content of particular items, that were tested. These findings, including illustrative quotes, are described in more detail in the results section.

### Factor analyses

All survey data analyses were conducted for each country separately, in Stata v15 [22]. We began by inspecting frequencies for responses to each item, to ensure items had enough variation across the four response categories (ie, from very dissatisfied to very satisfied).

We then conducted exploratory factor analysis (EFA) to explore the factor structure and item composition. In Mali, given a larger sample size, we split the sample randomly in half and conducted EFA on one half, and confirmatory factor analysis (CFA) on the other half in order to test the factor structure suggested by the EFA. In Bangladesh, we only conducted an EFA.

For the EFA in each country, we began by assessing the number of factors underlying the data by counting the number of eigenvalues >1, examining the scree plot (counting number of factors above the ‘elbow’) and conducting parallel analysis (counting the number of factors above the superimposed line). Based on this, we specified that number of factors, and used promax rotation since the factors were correlated. We also inspected one fewer, and one greater, than the number of factors, to help clarify how well items were loading on each factor. For each item, we examined: factor loading, with loadings <0.3 suggesting removal; uniqueness (inverse of communality), with values >0.7 or >0.8 suggesting removal; cross-loading (loading on more than one factor), also suggesting removal [23].

For the CFA, we tested the hypothesized factor structure resulting from the EFA. We first tested each factor (ie, scale sub-dimension) separately. We examined item factor loadings, with non-significant loading or loading <0.3 suggesting item removal, as well as model fit statistics including the root mean square error of approximation (RMSEA; with cutoff <0.08 indicating adequate fit), comparative fit index (CFI) and the Tucker-Lewis index (TLI) (cutoff >0.90 for both), and the standardized root mean square residual (SRMR; cutoff <0.08) [24]. We then added correlated error terms suggested by modification indices, and re-examined model fit [25].

We then tested a higher-order model in which the factors (scale sub-dimensions) just described loaded on a single higher-order factor (ie, Motivation). We confirmed adequate ‘factor loadings’ of each sub-dimension on the higher-order factor. We then added correlated error terms suggested by modification indices, and re-examined model fit.

### Reliability analyses

To assess reliability of the full scale and each sub-dimension, we calculated Cronbach’s alpha (with values of >0.7 indicating adequate fit and >0.8 good fit) [26], as well as Ordinal Theta, a measure of reliability similar to alpha but more suitable for the limited number of response categories (eg, 4) and scale items (eg, for the subdimensions) [27]. We also examined correlations between the factors as further evidence for a higher-order factor structure.

### Constructing final scale scores - weighting by importance

To generate a final score for each sub-dimension of Motivation, we calculated the mean of non-missing values for satisfaction items (each scored -2, -1, 1, 2 for very dissatisfied, dissatisfied, satisfied, very satisfied, respectively) for that sub-dimension. We then multiplied the mean score for each subdimension (which ranged from -2 to +2) by how important the respondent said it was to them, scored 1 = not very important, 2 = important, or 3 = very important. This resulted in a final sub-dimension score ranging from -6 to +6.

**Figure 1:**
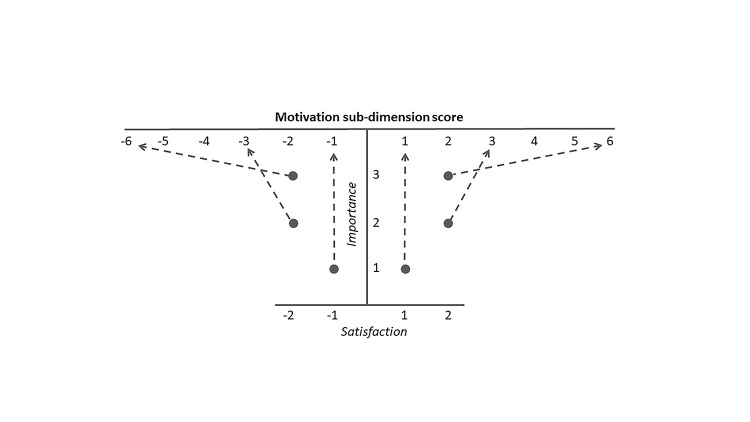
Visual illustrating the rationale for the scoring approach.

Of note, the negative (-2 -1)/positive (1 2) scoring for satisfaction items (and hence mean satisfaction score) was preferable to a positive scoring (eg, 1 2 3 4), since otherwise a low sub-dimension score for satisfaction multiplied by a high value on importance would result in a higher final score than if it was multiplied by a low value of importance. In other words, being unsatisfied with a dimension of your work, and considering that of high importance, should result in lower motivation compared with considering if of low importance. A visual representation of the rationale for this scoring is included in **Figure 1**.

Finally, to generate a motivation score for the full scale (ie, Motivation), we added up the sub-dimension scores, resulting in a final full-scale score ranging from -24 to +24 (since there were four final factors).

### Convergent validity analyses

To assess convergent validity – evidence of similarity between measures of theoretically related constructs [23]– we assessed associations between the scale scores (full and each sub-dimension) and conceptually-related constructs/variables. We also assessed associations between scale scores and performance-related variables (eg, self-reported number of household visits conducted; client-reported outcomes), as well as variables suggestive of retention (eg, years working as a CHW; reporting frequently thinking of quitting). This contributed to establishing predictive validity, evidence of the scale’s ability to predict future outcomes. Measures are described in **Table 1****.** Variables differed somewhat by country.

**Table 1:** Measures for variables used to assess convergent/predictive validity

Variable	Type of measure	Description
**Both countries**		
Overall interest in job	Ordinal, 4-level (Likert responses)	Response to question about satisfaction with overall interest in job, with response categories ranging from very dissatisfied to very satisfied.
Ability to improve health and well-being in your community	Ordinal, 4-level (Likert responses)	Response to question about satisfaction with ability to improve health and well-being in your community, with response categories ranging from very dissatisfied to very satisfied.
**Mali**		
Feels adequately supervised	Binary	Yes/no response to the question “Do you feel adequately supervised?”
Overall is motivated to work here	Ordinal, 5-level (Likert responses)	Response to the question “Overall I am motivated to work here.” Response categories included completely untrue, untrue, neutral, true, or completely true.
Performance:		
Number of household visits made in last month	Continuous	Self-reported number of household visits made in the last month.
Retention:		
Number of years working as a CHW	Continuous	Self-reported number of years working as a CHW.
Frequently thinks of quitting	Ordinal, 5-level (Likert responses)	Response to the question “I frequently think of quitting this job.” Response categories included completely untrue, untrue, neutral, true, or completely true.
**Bangladesh**		
Performance:		
Observed performance (index)	Continuous (range 0-1)	Mean performance score across observations of visits to multiple clients per CHW (mean of about 18 observations per CHW). Observed performance score is mean across scores relating to communication practices, FP method discussion, FP clinical check, and providing complete information. Six CHWs were missing this variable.
Quality of care reported by clients (index)	Continuous index (range 0-6)	Mean quality of care score across surveys with multiple clients per CHW (mean of about 20 clients per CHW). Quality of care score is sum of six yes/no items (example: “…were you told about side effects or problems you might have with the method?”). Six CHWs were missing this variable.
Client Trust in CHWs (scale)	Continuous (range 1-4)	Mean client trust score (10 item scale) across surveys with multiple clients per CHW (mean of about 20 clients per CHW). The Trust in CHWs scale includes two sub-dimensions: *Health care competence* and *Respectful communication*. Six CHWs were missing this variable. More information about this scale is described elsewhere (Sripad et al. [20]).
Client Empowerment in Community Health Systems (CE-CHS) Scale	Continuous (range 1-4)	Mean client empowerment score (16 item scale) across surveys with multiple clients per CHW (mean of about 20 clients per CHW). The Client empowerment scale includes three sub-dimensions: *Personal agency around health*, *Agency in sharing health information with others*, and *Empowerment in community health systems*. Six CHWs were missing this variable. More information about this scale is described elsewhere (McClair et al. [19]).
Retention:		
Number of years working as CHW	Ordinal, 3-level	Self-reported number of years working as a CHW: <5, 5 to 10, or >10 (original response categories; not recoded)

CHW – community health worker

We conducted multivariate regression analyses with the MM scale scores as the outcome; linear regression was employed in Mali; finite mixture modeling was employed in Bangladesh due to the bimodal distribution of motivation scores (as described further in the results section). We controlled for potential confounders, including gender (in Mali), age, education, marital status, number of years working as a CHW, and district/union. We chose to present multivariate rather than bivariate results to adjust for confounding of the true relationships between the variables of greatest interest.

### Ethics

This study was approved by the Institutional Review Board (IRB) at the Population Council (New York, NY, US; *p874* and *p864*). The study in Mali was also reviewed and approved by the ethical committee of the National Public Health Research Institute in Bamako, Mali (Le Comité d’Éthique de l’Institut National de Recherche en Santé Publique (INRSP); *n02013-1223/MS-SG*), and the Bangladesh study by the Bangladesh Medical Research Council *20608052019*. All participants provided written informed consent to participate.

## RESULTS

A total of 152 CHWs completed surveys in Mali, and 76 CHWs in Bangladesh. About forty percent of respondents in Mali, and all respondents in Bangladesh, were female. Over half of all respondents had completed more than a secondary-level education. Majority were married and over half lived in the community/village in which they work as a CHW. All respondents in Bangladesh, and nearly three-quarters in Mali, reported receiving financial compensation only. Other sample characteristics are included in **Table 2**. Bangladesh respondents were older and worked as CHW for longer than those in Mali.

**Table 2:** Sample characteristics, by country

	Mali (n = 152)		Bangladesh (n = 76)	
**n**	**%**	**n**	**%**
Gender – female	60	39.5%	76	100%
Age (years; mean, SD, range)	32.0 ± 7.3 (21-63)		46.2 ± 10.0 (24-58)	
Education (highest completed):				
-Less than secondary	29	19.0%	3	4.0%
-Secondary	37	24.3%	33	43.4%
-More than secondary	86	56.6%	40	52.6%
Married/cohabitating	122	80.3%	66	86.8%
Length of time working as community health worker (CHW)	Mean 6.0 years, (SD 2.6), (range 0-10)		<5 years: 4.0%, 5-10 years: 19.7%, >10 years: 76.3%	
Type of compensation:				
-Financial only	98	64.5%	76	100%
-Both financial and non-financial	54	35.5%	0	0%
Lives in village where works as a CHW	128	84.2%	43	56.6%
Type of CHW – family welfare assistant (vs family welfare visitor)	(n/a)	(n/a)	66	86.8%

**Table 3** includes item response frequencies and means for Satisfaction items (note only final scale items are presented). CHWs in both countries tended to report being satisfied or very satisfied with most items. In general, reported satisfaction was lower in Mali than in Bangladesh. Satisfaction was lowest for items related to compensation and workload in both countries, particularly in Mali. There was a notable degree of variation in endorsement of different items within the *Feeling valued and capacitated in your work* and *compensation and workload* sub-dimensions.

**Table 3:** CHW satisfaction items and response frequencies (for final scale items)

	Mali (n = 152)						Bangladesh (n = 76)						
**Scale sub-dimension**	**Strongly dissatisfied, %**	**Dissatisfied, %**	**Satisfied %**	**Strongly satisfied, %**	**Item mean (range -2 to +2) (SD)**	***Factor loading* † *(from Mali CFA, n = 76)***	**Strongly dissatisfied, %**	**Dissatisfied, %**	**Satisfied, %**	**Strongly satisfied, %**		**Item mean (range -2 to +2), (SD)**	
Quality of supervision* (6 items)						0.63							
Respect received from supervisors on performing well (1)	0.7	2.0	86.5	10.8	1.01 (0.50)	0.464 (0.63)	0	2.6	56.6		40.8		1.36 (0.63)
Support your direct supervisor gives you in your work (2)	0.7	5.4	83.8	10.1	0.97 (0.62)	0.810 (0.61)	0	2.6	63.2		34.2		1.29 (0.61)
Fairness with which your performance is measured (3)	0.7	4.7	89.2	5.4	0.94 (0.55)	0.722 (0.58)	0	2.6	69.7		27.6		1.22 (0.58)
Coordination between your supervisor, community health leaders and stakeholders (4)	0.7	7.4	88.5	3.4	0.86 (0.61)	0.698 (0.53)	0	1.3	68.4		30.3		1.28 (0.53)
Ease with which you can communicate with your supervisors (5)	0.7	7.4	81.8	10.1	0.93 (0.68)	0.760 (0.58)	0	1.3	50.0		48.7		1.46 (0.58)
Appreciation shown by your supervisor for your work (6)	0.7	4.1	91.2	4.0	0.94 (0.51)	0.637 (0.57)	0	1.3	56.6		42.1		1.39 (0.57)
**Feeling valued and capacitated in your work** (6 items)						0.95							
Respect received from community for doing this work (7)	0	5.9	80.3	13.8	1.02 (0.61)	0.361§	1.3	2.6	38.2	57.9		1.49 (0.76)	
Opportunities to update your health knowledge related to your work (8)	0	6.6	88.8	4.6	0.91 (0.55)	0.342	0	1.3	34.2	64.5		1.62 (0.56)	
Autonomy to make decisions while working (9)	0	5.9	92.1	2.0	0.90 (0.50)	0.609	0	5.3	64.5	30.3		1.20 (0.69)	
Opportunities to contribute your ideas to improve services (10)	0	8.6	88.2	3.3	0.86 (0.60)	0.966	0	0	60.5	39.5		1.39 (0.49)	
Consideration of your views and ideas by community health leaders and stakeholders (11)	0.7	8.6	88.8	2.0	0.83 (0.63)	0.838	0	0	67.1	32.9		1.33 (0.47)	
Availability of drugs, supplies and equipment for your work (12)	2.0	36.8	54.0	7.2	0.28 (1.10)	0.400	0	5.3	46.1	48.7		1.38 (0.75)	
**Peer respect and support** (5 items)						0.27‖							
Support your coworkers (CHWs) give you in your work (13)	0	1.3	86.2	12.5	1.10 (0.41)	0.675	0	0	57.9	42.1		1.42 (0.50)	
Attitude of colleagues (in our case, CHWs / peers) (14)	0	0.7	91.5	7.9	1.07 (0.32)	0.770	0	0	54.0	46.1		1.46 (0.50)	
Respect received from other CHWs on performing well (15)	0	1.3	87.5	11.2	1.09 (0.40)	0.751	0	0	54.0	46.1		1.46 (0.50)	
Cooperation amongst CHWs (16)	0.7	1.3	82.2	15.8	1.11 (0.51)	0.512	0	0	54.0	46.1		1.46 (0.50)	
Mutual trust CHWs have for each other (17)	0	3.3	84.9	11.8	1.05 (0.50)	0.685	0	0	52.6	47.4		1.47 (0.50)	
**Compensation and workload** (5 items)						0.37 §							
Amount of total financial incentives you receive (18)	42.8	46.7	10.5	0	-1.22 (0.50)	0.688	6.6	23.7	38.2	31.6		0.64 (1.32)	
Timeliness in receiving financial incentives (19)	38.8	59.9	1.3	0	-1.36 (0.56)	0.774	4.0	5.3	46.1	44.7		1.22 (0.99)	
Additional payment for your work (20)	11.8	74.3	13.2	0.7	-0.84 (0.83)	0.500	10.5	32.9	42.1	14.5		0.17 (1.32)	
Number of hours you work in a typical day (21)	0	8.6	90.8	0.7	0.84 (0.57)	0.343 ‡	4.0	17.1	68.4	10.5		0.64 (1.02)	
Long term job security (22)	7.9	55.9	34.2	2.0	-0.34 (1.09)	0.384 ‡	0	0	32.9	67.1		1.67 (0.47)	

CHW – community health worker

*In Mali, four respondents were missing responses for all items within the Quality of Supervision subdimension (n = 148)

†All factor loadings significant at the *P* < 0.001 level, except when indicated otherwise.

‡*P* < 0.01.

§*P* < 0.05.

*‖P <* 0.10.

### EFA results

In EFAs conducted with the pool of 32 candidate items in Mali (EFA sample) and Bangladesh, the number of eigenvalues >1, scree plot and parallel analysis indicated there were three or four latent factors in Mali, and three in Bangladesh. We tried three, four, and five factor solutions in each country. Certain items consistently performed poorly across factor solutions in one or both countries. Three items had factor loadings <0.3 and/or cross-loaded on multiple factors, and four others had high uniqueness (>0.7). (A list of dropped items is available from the authors upon request.) We therefore removed these seven items and re-ran EFAs. Four conceptually clear factors emerged, which we labeled *Quality of supervision, Feeling valued and capacitated in your work, Peer respect and support,* and *Compensation and workload*. The most consistent/cleanly loading sets of items in both countries were for the factors *Quality of supervision* and *Peer respect and support*. While most of the items eventually assigned to *Feeling valued and capacitated in your work* and *Compensation and workload* loaded clearly on those respective factors, some items had less consistent loadings; these few items were grouped with the dimensions we felt they fit best, for confirmatory testing in CFAs.

### CFA results

We tested the four factors emerging from the EFAs in CFAs in Mali. CFAs suggested all items had good factor loadings for *Quality of supervision* and *Peer respect and support*. For *Feeling valued and respected in your* work, we dropped two items (“Your personal safety while working in the community” and “Your ability to improve the health and well-being of the community”), and for *Compensation and workload* we dropped one item (“Amount of non-financial incentives you receive”) with low factor loadings and which caused inadequate model fit. We then re-ran CFAs for each sub-dimension with 22 total items. We added several correlated errors between items within each sub-dimension (as noted below in **Table 4**), and assessed final model fit, which was good (**Table 4**). For the higher-order CFA, after adding additional correlated error terms between items in different sub-dimensions, final model fit for the higher-order model was adequate. Final CFA factor loadings for each item, and loadings for each factor on the higher-order factor, are included in the middle column of **Table 3**.

**Table 4:** Final model-fit statistics – CFA (Mali, n = 76)*

	RMSEA (cutoff <0.08)	CFI (cutoff >0.90)	TLI (cutoff >0.90)	SRMR (cutoff <0.08)
Full scale (22 items) *(fit statistics for higher-order model)*	0.052	0.942	0.928	0.095
Sub-dimensions *(fit statistics for each sub-dimension model separately)*:				
-Quality of supervision (6 items)	0.047	0.992	0.985	0.038
-Feeling valued and capacitated in your work (6 items)	0.052	0.991	0.980	0.060
-Peer respect and support (5 items)	0.124	0.990	0.899	0.025
-Compensation and workload (5 items)	0.055	0.989	0.973	0.052

RMSEA – root mean square error of approximation, CFI – comparative fit index, TLI – Tucker-Lewis index, SRMR – standardized root mean square residual

*Number of correlated errors between items: 18 total. Quality of supervision – 1; Feeling valued and capacitated in your work – 2; Peer respect and support – 4; Compensation and workload – 1; Full scale – 10.

Correlations between the four sub-dimensions provided further support for the higher-order factor structure, although more so in Bangladesh than in Mali. Correlations ranged from 0.09 to 0.45 in Mali, and from 0.61 to 0.71 in Bangladesh (with the lowest values in each country corresponding to *Quality of supervision* with *Compensation and workload*, and the highest corresponding to *Quality of supervision* with *Feeling valued and capacitated in your work*).

### Reliability

Internal consistency reliability is presented in **Table 5**. Cronbach’s alpha for the full scale was 0.84 in Mali (0.94 for Ordinal Theta) and 0.93 in Bangladesh. All reliabilities for sub-dimensions, for which Ordinal Thetas are reported (where calculable) given limited number of items, were >0.7; most were >0.8.

**Table 5:** Scale reliability

	Mali (n = 152)		Bangladesh (n = 76)	
**Alpha**	**Ordinal theta**	**Alpha**	**Ordinal theta**
Full scale (22 items)	0.84	0.94	0.93	–*
Sub-dimensions:				
-Quality of supervision (6 items)	0.91	0.95	0.92	0.96
-Feeling valued and capacitated in your work (6 items)	0.73	0.91	0.83	–*
-Peer respect and support (5 items)	0.84	0.95	0.98	–*
-Compensation and workload (5 items)	0.67	0.80	0.60	0.70

*Unable to calculate due to missing values in polychoric correlation matrix.

### Assessing importance to CHWs of each factor

**Table 6** presents CHWs’ reports of how important each sub-dimension was to them (asked via reading out a composite statement describing the sub-dimension, in its entirety). In Mali, a majority answered “very important” for *Quality of supervision* and *Compensation and workload*, and “important” for the other two sub-dimensions. In Bangladesh, a large majority answered “Very important” for each of the sub-dimensions.

**Table 6:** Importance to CHWs of different dimensions of their work (corresponding to sub-dimensions)

	Mali (n = 152)			Bangladesh (n = 76)		
**Importance of the following aspects of work**	**Not very imp. (%)**	**Imp. (%)**	**Very imp. (%)**	**Not very imp. (%)**	**Imp. (%)**	**Very imp. (%)**
**Quality of supervision** (that is, your supervisor(s) actively support and value your work, and treat you respectfully and fairly)	0	36.5	63.5	1.3	5.3	93.4
Feeling valued and capacitated in your work*† (that is, feeling your work is respected by the community, you have the training and tools you need, and can make decisions and have your ideas considered)	0	65.8	34.2	0	7.9	92.1
**Peer respect and support** (that is, support, trust and cooperation among your peers at work)	4.0	74.3	21.7	1.3	6.6	92.1
Compensation and workload* (that is, enough and timeliness of compensation, including in relation to your workload)	0	31.6	68.4	2.6	5.3	92.1

CHW – community health worker

*Original items were worded as follows: Growth opportunities (that is, having enough opportunities to improve your professional skills, make your own decisions, and have your ideas considered); Compensation (that is, sufficient amount and timeliness of incentives, including in relation to your workload and the time you spend with your family).

†3 of 5 items in the final “Feeling valued and capacitated in your work” sub-dimension (**Table 2**) originally fell under “Growth opportunities”, which is why this importance item was used in the present analyses.

### Qualitative findings relating to motivation and its sub-dimensions

In both countries, CHWs who participated in the qualitative research recognized that they play critical roles in their communities and take pride in their work. Qualitative data in each country supported the relevance of each sub-dimension to motivation. While there was some consistency in how satisfied CHWs were with the different dimensions of their work, this also varied to some extent- particularly with respect to ‘extrinsic’ factors like amount of incentives or training received. Illustrative quotes for each sub-dimension are included in **Table 7**.

**Table 7:** Illustrative quotes relating to Motivation scale sub-dimensions

DIMENSION	Mali focus group discussions with Community Health Workers	Bangladesh in-depth interviews with Family Welfare Assistants (FWAs) and supervisors
**Quality of supervision**	“*[The supervisors] support us in our work and guide us. I find that supervision is important…it allows us to better understand certain aspects in order to improve*.” (Site 1)	“*Our supervisor helps us a lot. Whenever we face any problem, he helps*.” (FWA 02)
	“*Really with my DTC [supervisor] it is mutual respect. He calls me often to ask about my availability, and he asks if I have problems; if yes I explain*.” (Site 4)	“*The training is important to me. I think it helps me in doing my job better. Supervisors regularly take care of me which is also important*.” (FWA 01)
**Feeling valued and capacitated in your work**	*Facilitator*: “*Do the villagers value you in your work*?” *Participant*: “*They do because it’s important…every time you explain things to them in your chat sessions…they really listen to us*.” (Site 4)	“*The respect [the community] gives us, it encourages us. Even though we are not a doctor, they treat us as one when we go there*.” (FWA 05)
	“*We don’t have work tools, we always tell our [supervisors], and they too tell their supervisors…if you want take care of someone, you need work tools otherwise it’s not easy*.” (Site 7)	“*People are getting care through me, they rely on me, they trust me...[this] makes me happy. I have reached them through my work*.” (FWA 04)
		*“We have some books, leaflets, pictures, flipchart…those help us a lot.”* (FWA 01)
		“*The supply of medicines is not enough. We can’t fulfill the demand…*” (FWA 07)
		“*Training of the workers is essential. [But] it has been a long time without training, not even once or twice in a year*.” (Supervisor 01)
**Peer respect and support**	“*Between us we have no difficulty, because when you call a colleague and ask him for an explanation, he explains to you ... we support each other*.” (Site 1)	“*We cooperate. In the community health care system, we give condoms, pills, other methods...we [also] refer them to the [family welfare] visitor …We provide all the services so they can get good treatment*.” (FWA 03)
		“*We organize camps in different places…[but sometimes they create religious obstacles]…Then we try to make them understand.*” (Supervisor 03)
**Compensation and workload**	“*The difficulty we have is the lack of money, when the wages do not come on time we can no longer work as well*.” (Site 5)	“*Government is paying salary for us as well as compensation for all clients who are taking Copper-T, implant, ligation and NSV. I will be happy if the amount of compensation increases*.” (FWA 10)
	“*Really [the compensation] is not enough - it doesn’t allow me to support myself. If they could increase it would help us a lot.” “It is our wish that they increase it. The problem is also that it doesn’t come every month, and as I am the head of the family, we cannot stay like that.”* (Site 4)	“*If I had less workload, had to write less, I could continue my work more easily and* *comfortably alongside my family work.”* (FWA 06)

*Quality of supervision*, in terms of both ‘soft’ skills such as being friendly and respectful, and ‘harder’ skills like assisting in-person when problems arose, was described as critical to supporting CHWs to do their job well, including in relation to facing problems in the community, as well as finding solutions to reaching targets. In Mali, where CHWs had fewer years of experience than in Bangladesh and cover a range of health areas, they appreciated that supervisors engaged in a constant manner, in person and via phone, to answer questions and address problems as they arose.

*Feeling valued and capacitated in your work* was described in terms of feeling valued in the community and feeling valued and capacitated by the community health system, the latter relating both to ideas and autonomy as well as adequate training and supplies (eg, job aides, medicines).

*Peer respect and support* was consistently described in the interviews; in Bangladesh this was related both to mutual support between FWAs (ie, the community-based CHWs), and between FWAs and FWVs (the facility-based CHWs), and was seen as particularly important when confronted with community or religious pushback to family planning services.

*Compensation and workload* were described as important to staying motivated, and evaluated differently in relation to salary vs other forms of compensation such as travel reimbursements or incentive-based payments. Timeliness of compensation was described as a problem in Mali, but not in Bangladesh. Also in Mali, job security was perceived as threatened since they work for a nongovernmental organization (NGO) on a non-regular basis, rather than for the government.

Finally, CHW and supervisors described certain contextual circumstances as limiting their ability to do their job well. Examples in Bangladesh were the high mobility of clients, particularly in urban settings, and residual cultural norms against family planning, and in Mali, the periodic political instability. CHWs seemed to see these factors as relatively immutable, and hence seemed to downplay them as central to their sense of motivation in their work.

### Final scale scores (weighted by importance)

Descriptive statistics for the final scale scores are included in **Table 8**. The mean scores for the full scale and for each sub-dimension were lower in Mali than in Bangladesh. *Compensation and workload* was the sub-dimension with the lowest mean score in both countries, and had a negative value in Mali. The highest mean score was for *Quality of supervision* in Mali, and for *Peer respect and support* in Bangladesh. In Mali, scores did not differ significantly by the CHW’s gender (data not shown).

**Table 8:** Final Motivation scale scores

	Mali (n = 152), mean score (SD)	Bangladesh (n = 76), mean score (SD)
Full scale (Possible range: -24 to +24)	4.95 (3.28)	14.51 (5.29)
Sub-dimensions (Possible range for each: -6 to +6):		
-Quality of supervision	2.47 (1.45)	3.93 (1.47)
-Feeling valued and capacitated in your work	1.86 (1.17)	4.11 (1.36)
-Peer respect and support	2.35 (0.92)	4.27 (1.57)
-Compensation and workload	-1.68 (1.45)	2.20 (1.83)

SD – standard deviation

**Figure 2:**
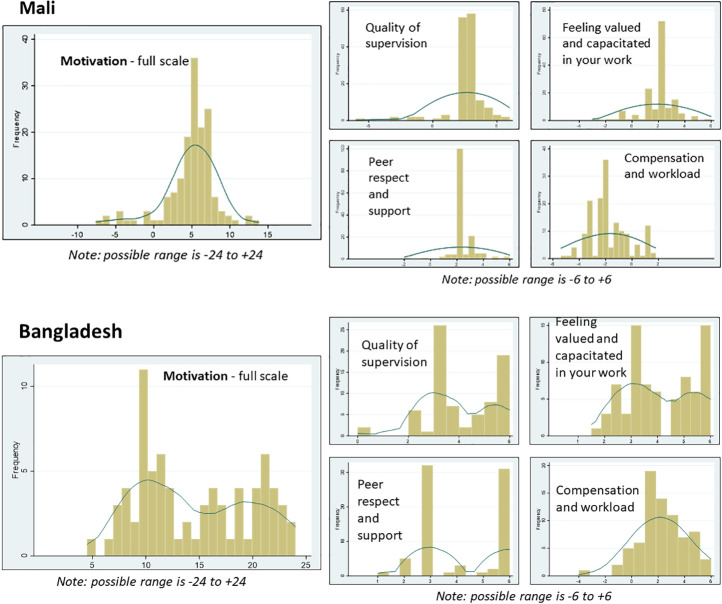
Scale score distributions.

The distributions of scale scores are presented in **Figure 2**, using histograms overlaid with smoothed kernel density estimates. In Mali, the scores were normally distributed and were somewhat skewed towards higher scores (except for *Compensation and workload)*. In Bangladesh, with the exception of the *Compensation and workload* which was normally distributed, the full scale and other sub-dimensions each had a bimodal distribution, consisting of two distinct ‘normal’ distributions, one with lower values and one with higher values. We conducted ancillary analyses to try to identify factors that may be causing the bimodal distribution in Bangladesh, but found that none of the following factors were associated (in finite mixture models, as described in more detail below): CHW cadre (FWA vs FWV), years working as a CHW, age, education, and union.

### Convergent and predictive validity

Results of multivariate regression analyses are presented in **Table 9** for Mali and **Table 10** for Bangladesh. In Bangladesh, due to the bimodal distribution of the full scale as well as the sub-dimensions (except for *Compensation and workload*), we used finite mixture modeling (via the “fmm 2” command in Stata) to generate two coefficients, one for the distribution with lower values, and one for the distribution with higher values (**Figure 2**). Control variables are noted below each table.

**Table 9:** Mali – multivariate regression results (n = 152)

		Motivation sub-dimensions (range -6 to +6)			
	**Motivation full scale (range -24 to +24)**	**Quality of supervision**	**Feeling valued and capacitated in your work**	**Peer respect and support**	**Compensation and workload**
	Adjusted* **beta coefficients** (Motivation/subdimensions as outcome)				
Overall interest in job	**4.01**§	1.31§	1.08§	0.37†	1.20§
Ability to improve health and well-being in your community	**4.18**§	1.46§	1.25§	0.89§	0.45
Overall is motivated to work here	**3.37**§	1.70§	0.95§	0.15	0.57†
Feels adequately supervised (binary)	**4.24**§	1.37§	1.04§	0.36	1.37§
**Suggestive of performance:**					
Number of household visits made in last month‖ (continuous; self-reported)	**0.09**†	0.07§	0.02	0.00	-0.01
**Suggestive of retention:**					
Number of years working as a CHW (continuous)	**-0.19**	-0.06	-0.07	0.00	-0.06
Frequently thinks of quitting	**-1.11**‡	-0.61‡	-0.26	0.05	-0.36†

*Analyses controlled for gender, age, education, marital status, district, and number years working as CHW.

†*P* < 0.05.

‡*P* < 0.01.

§*P* < 0.001.

‖Also controlled for number of hours works per week.

**Table 10:** Bangladesh – multivariate regression results (n = 76)

		Motivation sub-dimensions (range -6 to +6)			
	**Motivation full scale (range -24 to +24)**	**Quality of supervision**	**Feeling valued and capacitated in your work**	**Peer respect and support**	**Compensation and workload**
	Adjusted beta coefficients for 1st and 2nd distributions, given bimodal distributions (except for compensation and workload, for which beta is from linear regression)*				
Overall interest in job (range 1-4)	**2.27**†, **11.05**§	-0.29, 2.65§	0.34§, 2.65§	(did not converge)	1.25‡
Ability to improve health and well-being in your community (range 1-4)	**0.24, 8.27**§	-0.35, 2.77§	0.53‡, 2.90§	(did not converge)	1.31§
Performance:‖					
Observed performance (mean score across clients, range 0-1)	**-1.54, -6.86**	1.20, 2.53§	4.00§, 1.98	0.06, 2.55‡	-2.83
Quality of care reported by clients (mean score across clients, range 0-6)	**0.50, -1.47**†	-0.22, -0.01	0.28, -0.48†	0.03, 0.24‡	-0.36
Client Trust in CHWs (mean score across clients, range 1-4)	**0.73, -2.25**	2.03§,1.79†	-0.96†, 0.30	(did not converge)	-0.07
Client Empowerment (mean score across clients, range 1-4)	**1.73 §, 0.54**	0.39 §, 0.46	1.22 †, -0.94	(did not converge)	0.04
Suggestive of retention:					
Number of years working as CHW (<5, 5 to 10, >10)	**17.06§, -5.82**†	2.29†,0.56	-0.79†, 1.40‡	0.05, 0.38	-0.67

*The first beta represents the association among the class scoring lower on Motivation/the sub-dimension; the second beta is among the class scoring higher on Motivation/the sub-dimension. Analyses controlled for age, education, marital status, union where CHW works, and number years working as CHW.

†*P* < 0.05.

‡*P* < 0.01.

§*P* < 0.001.

‖n = 62 for these analyses, given 6 missing on performance measures, plus 8 additional removed from analysis because FWVs (given less applicable than FWAs).

In both countries, the full scale as well as each sub-dimension were positively and significantly associated with two items reflecting general work satisfaction/motivation: “Overall interest in job” and “Ability to improve health and wellbeing in your community”. In Mali, the full scale and sub-dimensions were also associated with a general assessment of motivation “Overall is motivated to work here” as well as reporting feeling adequately supervised (these two items were not included in the Bangladesh survey).

Turning to associations with variables suggestive of performance (controlling for potential confounders), in Mali a higher score on the full motivation scale was associated with visiting a larger number of households in the last month, as was the *Quality of supervision* sub-dimension. In Bangladesh, a higher score on the full scale and/or sub-dimensions was associated with several indicators of performance. These associations varied somewhat based on whether the CHW fell within the lower vs higher distribution of motivation. For observation-based performance, higher motivation around *Quality of supervision* and *Peer respect and support* was associated with higher performance among CHWs in the higher distribution; higher motivation around *Feeling valued and capacitated in your work* was associated with higher performance among those in the lower distribution. For client-reported quality of care, *Peer respect and support* was associated with higher quality of care but *Feeling valued and capacitated in your work* with lower quality of care, among CHWs in the higher distribution. Finally, among CHWs in the lower distribution, a higher score on the full motivation scale was associated with higher client-reported empowerment, *Quality of supervision* was associated with higher empowerment as well as trust in CHWs, and *Feeling valued and capacitated in your work* was associated with higher trust in CHWs.

Finally, for associations with variables suggestive of retention, in Mali, a higher score on the full motivation scale was inversely associated with frequently thinking of quitting, as were the *Quality of supervision* and *Compensation and workload* sub-dimensions, but there were no associations with number of years working as a CHW. In Bangladesh, there was a strong association between a higher score on the full motivation scale and number of years working as a CHW, but only for CHWs on the lower distribution of motivation.

**Figure 3:**
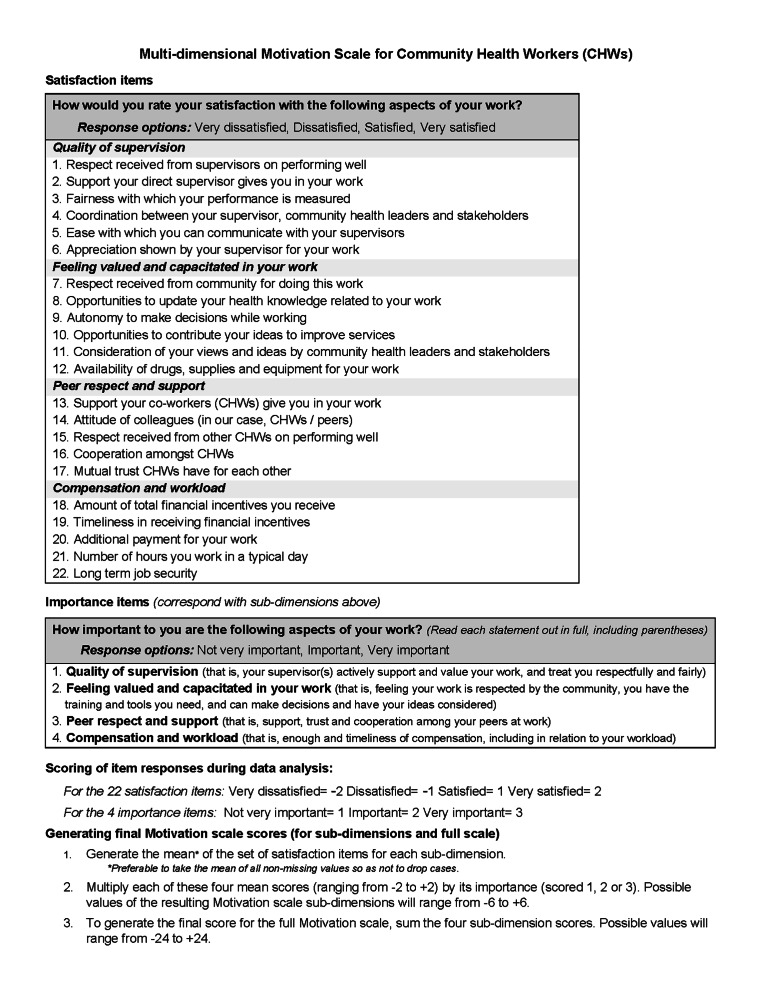
Multi-dimensional Motivation Scale items and scoring instructions.

**Figure 3** presents the final Multi-dimensional Motivation (MM) Scale, including items and scoring instructions.

## DISCUSSION

We found that the new Multi-dimensional Motivation (MM) scale among CHWs was valid and reliable and we encourage its use to track and evaluate interventions to improve CHW motivation. While perhaps unconventional, we believe the two-step approach of assessing key dimensions of satisfaction, then weighting each by its importance, operationalizes the construct motivation in a pragmatic way. The scale demonstrated very good psychometric properties, as well as associations with other related variables, including indicators of performance and retention. The final four scale sub-dimensions align well with previous qualitative research with CHWs in other contexts as well as our own qualitative research [9,13,16].

The final scale sub-dimensions were similar to those we had hypothesized, particularly for *Quality of supervision, Peer respect and support,* and *Compensation and workload*. The *Feeling valued and capacitated in your work* sub-dimension includes a combination of items from three other originally hypothesized dimensions. In fact, recent multi-country qualitative research has shown that characteristics like feeling respected by the community, having agency in decision-making, and having the tools/resources to do your job often form a single theme around feeling valued [9].

Mean levels and distributions of motivation varied substantially by country, with the mean scores for the full scale and each sub-dimension being higher in Bangladesh than in Mali. This could owe to the more mature community health system in Bangladesh including a formal cadre of CHWs who are regularly compensated for their work. In comparison, the CHWs in Mali were contracted by an NGO, leading to uncertain job security and income (as noted in qualitative interviews) due to fluctuations in funding availability. While there were relatively high mean scores in Bangladesh, it does not appear to have a ‘ceiling effect’ – that is, the distribution is not too highly skewed towards high motivation – at least in Mali and Bangladesh, unlike another recent scale related to CHW motivation (that was administered in Ethiopia, Kenya, Malawi and Mozambique) [18]. The distribution was bimodal in Bangladesh, suggesting that there may be two distinct motivation experiences among CHWs. It remains unclear why there is a bimodal distribution; no logical underlying factors (eg, CHW cadre, years as a CHW, union, age, education) appeared to explain it. We recommend future implementers and researchers pay close attention to the distributions of the full scale and its sub-dimensions, as well as reasons for any non-normal distributions.

The level of importance CHWs assigned to each dimension also differed between the two countries. In Mali, two sub-dimensions (*Feeling valued and capacitated in your work* and *Peer respect and support*) were seen by a majority as “important” vs “very important”, whereas in Bangladesh nearly all respondents reported that all sub-dimensions were very important. It may be less critical to weight the satisfaction sub-dimensions by importance in contexts like Bangladesh where CHWs see all dimensions as similarly important (given that doing so would have a minimal effect on variation of the final score). However, we believe it is still useful to assess relative importance in surveys and recommend following the weighting/scoring protocol recommended in this paper for comparability across contexts.

We believe the scale is likely to perform well in other contexts. Item content and wording is intended to be applicable to any geographic context, cadre of CHWs, and health area. We also based scale sub-dimensions/item content on research findings from multiple countries and types of CHW programs [9,13-16]. While we evaluated the scale in only two countries, they are in two different regions of the world with distinct socio-cultural contexts and community health systems, and varied scopes of work for CHWs. Still, the scale will benefit greatly from continued evaluation in different contexts.

There are several implications findings for use of this newly validated scale in future research and community health systems strengthening efforts. The scale provides an in-depth understanding of motivation among CHWs, and can be used to explore differences by relevant subgroups as well as changes over time. Longitudinal research is needed to further understand the scale’s ability to predict outcomes of interest, as well as changes in these outcomes based on an intervention. In any research around CHW motivation and performance, as well as intervention effects, it is critical to assess the influence of contextual factors such as health system policy and practice, safety and security, and socio-cultural factors, among others [28,29]. Our qualitative findings in Mali and Bangladesh suggested that both health system and societal-level factors fundamentally shape CHWs’ work experiences and well-being.

The MM scale captures several of the components put forth by World Health Organization on the recent guidelines on health policy and system support to optimize CHW programs [2] and could be used to monitor global recommendations around the need for countries to document effects of community health systems strengthening strategies including CHW selection, pre- and in-service trainings, certification, renumeration, and career development. The MM scale could also complement studies on CHW job preferences such as discrete choice experiments [30,31]. The MM scale may be feasible to integrate into in national CHW surveys [32], special studies, and/or community health roadmap monitoring strategies [2,33].

This study has several limitations. First, we evaluated the new scale in relatively small samples in two countries. However, findings about item performance and factor structure, item performance, and scale performance were quite similar in the two countries, and were further reinforced by qualitative findings. Second, sampling of CHWs for participation in surveys was non-random, potentially leading to selection bias. Third, we conducted qualitative research in parallel with scale development and refinement, rather than prior to it as is preferable [23]. Fourth, due to the cross-sectional nature of survey data, convergent and predictive validity analyses cannot demonstrate causality; as noted previously longitudinal studies are needed. Finally, the generalizability of findings within the two countries, other countries and CHW cadres, or other health areas (eg, HIV/TB) is not assured. Confidence in applicability of the scale across varied contexts is increased for the reasons noted above.

## CONCLUSION

The availability of multidimensional measures of motivation among CHWs is essential to community health systems research and community health services management. These measures should be valid and reliable, as well as pragmatic and applicable across contexts. Findings from this study show that the MM scale meets these criteria. We hope that this new scale, alongside others developed as part of the Frontline Health Project, will have a positive impact on supporting CHWs and strengthening community health systems worldwide.
